# Proteomic analysis of tea plants (*Camellia sinensis*) with purple young shoots during leaf development

**DOI:** 10.1371/journal.pone.0177816

**Published:** 2017-05-16

**Authors:** Qiongqiong Zhou, Zhidan Chen, Jinwook Lee, Xinghui Li, Weijiang Sun

**Affiliations:** 1 Anxi College of Tea Science, Fujian Agriculture and Forestry University, Fuzhou, China; 2 College of Horticulture, Henan Agricultural University, Zhengzhou, China; 3 Department of Horticultural Science, Mokpo National University, Muan, Republic of Korea; 4 Tea Research Institute, Nanjing Agricultural University, Nanjing, China; Universidade de Lisboa Instituto Superior de Agronomia, PORTUGAL

## Abstract

Tea products made from purple leaves are highly preferred by consumers due to the health benefits. This study developed a proteome reference map related to color changes during leaf growth in tea (*Camellia sinensis*) plant with purple young shoots using two-dimensional electrophoresis (2-DE). Forty-six differentially expressed proteins were detected in the gel and successfully identified by using MALDI-TOF/TOF-MS. The pronounced changes in the proteomic profile between tender purple leaves (TPL) and mature green leaves (MGL) included: 1) the lower activity of proteins associated with CO_2_ assimilation, energy metabolism and photo flux efficiency and higher content of anthocyanins in TPL than those in MGL may protect tender leaves against photo-damage; 2) the higher abundance of chalcone synthase (CHS), chalcone isomerase (CHI) and flavonol synthase (FLS) likely contributes to the synthesis of anthocyanins, catechins and flavonols in TPL tissues; 3) higher abundance of stress response proteins, such as glutathione S-transferases (GST) and phospholipid hydroperoxide glutathione peroxidase (PHGPx), could enhance the tolerance of TPL tissues to adverse condition in; and 4) the increased abundance of proteins related to protein synthesis, nucleic acids and cell wall proteins should be beneficial for the proliferation and expansion of leaf cell in TPL tissues. qPCR analysis showed that the expression of differentially abundant proteins was regulated at the transcriptional level. Therefore, the results indicated that higher abundance of CHI and CHS may account for the production of the purple-shoot phenotype in Wuyiqizhong 18 and thereby, enhancing the anthocyanin biosynthesis. The higher abundance of glutamine synthetase (GS) proteins related to the theanine biosynthesis may improve the flavor of tea products from TPL materials. Thus, this work should help to understand the molecular mechanisms underlying the changes in leaf color alteration.

## Introduction

Tea (*Camellia sinensis* (L.) O. Kuntze) is an important commercial crop that is grown mostly in the tropical and subtropical countries of Asia, Africa and to some extent Latin America [1]. In recent years, tremendous attention has been focused on tea plant due to its pleasant flavor and bioactive substances. Tea leaves are a valuable source of secondary metabolic products, including flavonoids, alkaloids, polysaccharides and theanine [2]. Among these secondary metabolites, flavonoids (which comprise polyphenols, flavones, flavanonols and anthocyanins) are considered to contribute numerous pharmacological beneficial effects on human health [3–4]. The health benefits of tea are thought to account for tea’s protective role against cardiovascular disease [3], atherosclerosis [4], oxidant activity [5], and cancer [6].

The color of tea leaves has been diversified through long-time natural hybridization and artificial selection. Study on purple leaves of tea plant will positively promote the diversification of tea products and the fully utilization of different tea germplasm resources. Furthermore, tea products made from purple tea leaves are highly preferred by consumers. Compared with conventional tea, the anthocyanin-rich purple tea has multiple benefic functions including strong antioxidant activity [7], inhibition of colorectal carcinoma cell proliferation [8], and reinforcing brain antioxidant capacity [9]. In addition, anthocyanins can be used as commercial food colorants and have been reported to exhibit antioxidants, chemopreventive, anti-bacterial, antiangiogenic, anti-inflammatory and anti-atherosclerotic properties [10]. Therefore, to enhance the health potency of tea, purple-colored leaves have become one of the main qualitative attributes targeted in tea breeding programs. New tea cultivars with purple leaves have recently been developed and released in Kenya, China, Japan and India [11, 12–14].

The contents of total phenolic compounds and total anthocyanins, including catechins were higher in purple tea leaves than in green tea ones [15, 16]. Previous study has found that malvidin is the most abundant anthocyanidin in the tea products derived from the purple colored tea clones [11]. Anthocyanins often accumulate in young leaves of plants (including tea plants), and make leaves present a purple color, then the purple color in leaves gradually turn into green as the leaves mature [17]. Additionally, anthocyanin in young leaves correlates with tolerance to photoinhibition by the attenuation of visible radiation. Maayan [18] have reported that ribulose-1,5-bisphosphate carboxylase/oxygenase (rubisco), as well as chlorophyll, is not present in early development stages and only slowly accumulates throughout the developmental stages of the leaves. The lower abundance of rubisco is closely related to the rate of photosynthesis in young purple leaves [19]. In previous study, we used cDNA-AFLP to investigate the gene expression profiles associated with changes in leaf color during the developmental stages of tea leaves, and 148 differentially expressed genes, which were involved in cell respiration, nucleic acid and protein metabolism, photosynthesis, cell expansion, biosynthesis of flavonoids and anthocyanins, were examined [19]. However, the identification of differentially expressed genes at mRNA level is insufficient for the prediction of protein expression levels, and the available proteomics information on the changes in metabolites during shoot development in purple-leaf-tea cultivars is limited. Therefore, in this study, we used the newly developed tea cultivar Wuyiqizhong18 as source material, and we conducted 2-DE to separate differentially abundant proteins at the proteomic level and characterize the molecular mechanisms underlying changes in leaf color during the growth of tea plants with purple young shoots.

## Materials and methods

### Plant materials

**Fig 1:**
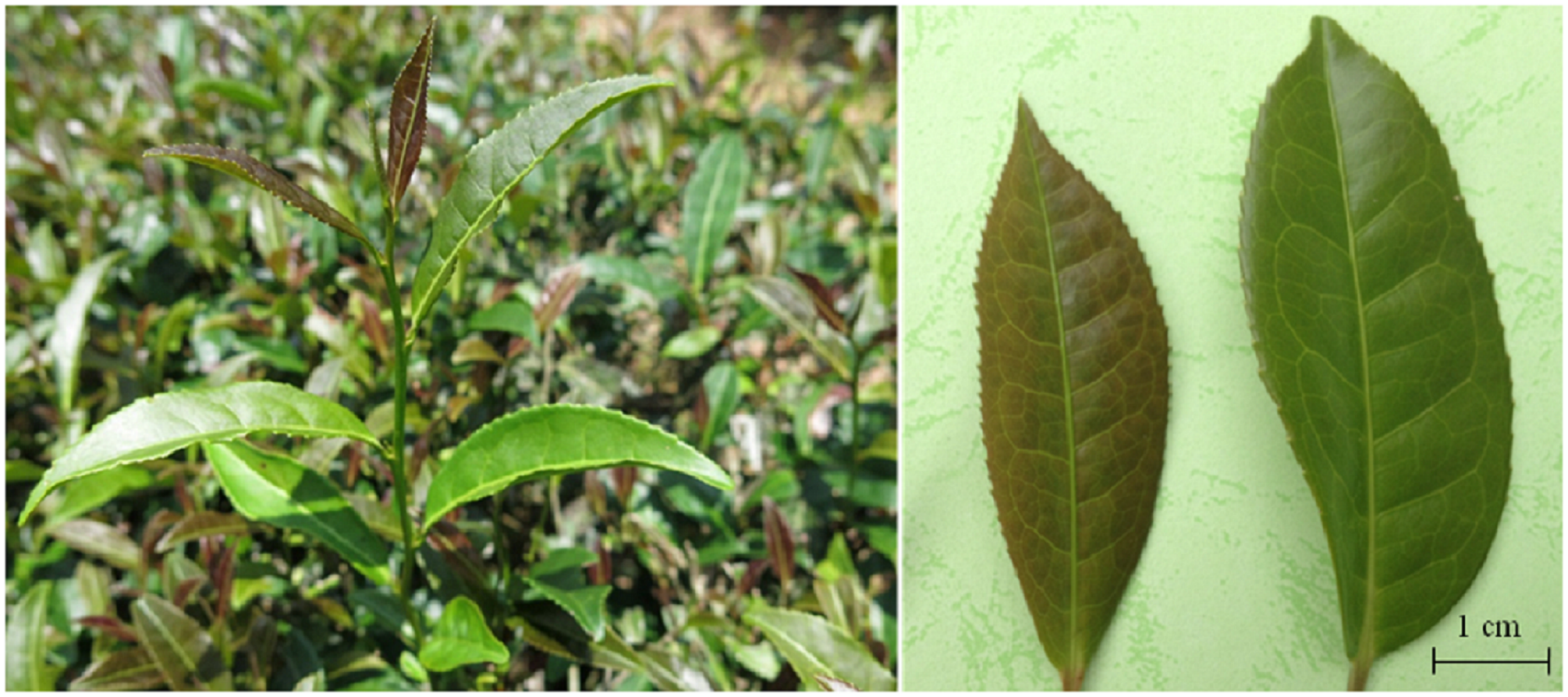
The tender purple (TPL) and mature green leaves (MGL) of Wuyiqizhong18 tea plant.

Tea plants (*C*. *sinensis* (L.) O. Kuntze cv.Wuyiqizhong18) were grown in the Germplasm Repository of Tea Research Institute in Wuyi Mountain, Fujian province, China. Only the healthy, tender shoots (one bud and two leaves) were sampled on April 13, 2015, and then the purple and green leaves were packaged separately in aluminum foil. The plant characteristics in ‘Wuyiqizhong18’ tea cultivar were as follows: the bud were green, the color of the second leaf changes gradually to purple, and the leaves finally turned green again (Fig 1). The samples were immediately frozen in liquid nitrogen and stored at −80°C until use.

### Measurement of polyphenolic compounds

Anthocyanins were extracted with 1% HCl-methanol solution and measured using a spectrophotometer at 530 nm, according to method described by Hatlestad et al. [20]. The contents of total polyphenols were determined according to the International Organization for Standardization (ISO) 14502–1:2005 using Folin—Ciocalteu method [16]. The identification and quantification of catechins and caffeine were measured according to the method described by Wei et al. [21].

### Protein extraction

Total protein was extracted from frozen leaf samples using a phenol extraction procedure according to Saravanan et al. [22] with some modifications. Briefly, about 1 g leaf sample was ground into fine powder in liquid N_2_ with a pestle and mortar. Approximately, 4 mL of ice-cooled extraction buffer [100 mM Tris-HCl (pH 7.8), 50 mM L-ascorbic acid, 1% (v/v) Triton X-100, 1% (v/v) β-mercaptoethanol, 100 mM KCl, and 1 mM phenylmethylsulfonyl fluoride] was added to the powder. The mixture was allowed to thaw slowly at room temperature and then gently pulverized for another two minutes. The homogenate was transferred to 10 ml centrifuge tube and centrifuged at 13,000 g for 15 min at 4°C after equal volume of Tris-saturated phenol (pH 8.0) was added. The phenolic phase was transferred to a 50 mL corning centrifuge tube with five volumes of methanol (with 100 mM ammonium acetate). The protein pellets were collected and re-suspended in 25 mL of ice-cooled pure methanol for 2 h at -20°C after centrifugation at 13,000 g for 10 min at 4°C. Protein pellets were collected by centrifugation at 13,000 g for 15 min at 4°C and washed twice with 25 mL of ice-cooled acetone, and then lyophilized at -20°C until use. About 10 mg protein pellet was dissolved in lysis buffer [8 M urea, 1.5 M Thiourea, 4% (w/v) CHAPS, 2% (v/v) ampholyte (3–10), 15 mM DTT]. The total protein concentration was measured by using Bradford protein assay [23].

### 2-DE and image analysis

Protein samples (about 1.5 mg) were rehydrated with a rehydration buffer containing 8 M urea, 2 M thiourea, 4% (w/v) 3-[(3-cholamidopropyl)-dimethylammonio] propanesulfonate (CHAPS, 0.5% (v/v) IPG buffer, 13 mM dithiothreitol (DTT), and 0.002% (w/v) bromophenol blue, then loaded onto a 24 cm, pH 4–7 linear immobilized pH gradient (IPG) strip (GE Healthcare, Uppsala, Sweden) on the rehydration cassette overnight. Isoelectric focusing electrophoresis (IEF) was conducted according to the methods described by You et al. [24].

Image analysis was carried out with PDQuest software version 8.0.1 (Bio-Rad, Hercules, CA, USA). Spot detection, matching and normalization were performed automatically, followed by manual verification. Only those candidate spots in all triplicate gels which had both a fold change of more than 1.5 and a *p* value of less than 0.05 by statistical test were considered differentially abundant proteins for mass spectrometry analysis.

### Protein identification by MALDI-TOF/TOF-MS and bioinformatics analysis of proteins

Protein spots were excised from the stained gels and digested with trypsin as described by You et al. [24]. Mass spectrometric analysis was conducted with a MALDI-TOF/TOF-MS 5800 (AB SCIEX, Shanghai, China). All acquired full-scan MS of samples were processed using TOF/TOF Explorer™ Software (AB SCIEX) in—default mode. The data were analyzed through the GPS Explorer software (Applied Biosystem, Foster City, CA, USA) using MASCOT software (Matrix Science Inc., MA, USA) with the database viridiplantae from NCBI website (1850050 sequences; 642453415 residues). The search criteria specified trypsin, allowing 1 missed cleavage, with a peptide tolerance of 100 ppm of and an MS/MS tolerance of 0.6 Da. Proteins exhibiting a score greater than 75 and statistical significance (*p* < 0.05) were considered to be successfully identified proteins and were accepted.

Functional annotation of proteins was conducted using Gene Ontology (GO) annotation [25]. The differentially accumulated proteins were assigned to the Cluster of Orthologous Groups of proteins (COG) database and the Kyoto Encyclopedia of Genes and Genomes (KEGG) database [26].

### qPCR analysis

Total RNA extraction and qPCR were performed as previously described [19]. Specific primers were designed to investigate the gene expression at the transcript level of these representative differentially accumulated proteins. The sequences of the forward (F) and reverse (R) primers were listed in S1 Table. For the normalization of gene expression, *Camellia sinensis* 18S ribosomal RNA (AB120309.1) was used as a reference gene. Each treatment contained 3 biological replicates and each biological replicate had 3 technical replicates. The specificity of each primer pair was verified by melting curve analysis. The relative gene expression was calculated using the 2^-ΔΔCt^ algorithm below [27].

$$\Delta \Delta Ct={\left(Ct_{gene\;of\;interest}-Ct_{internal\;control}\right)}_{treatment}-{\left(Ct_{gene\;of\;interest}-Ct_{internal\;control}\right)}_{control}$$

### Statistical analysis

Significance tests were conducted using Student’s t-test at *p* < 0.05 level. The results are represented by the mean ± standard errors (SE).

To elucidate the overall proteomic responses of tea leaves, we performed a heatmap approach by using mean centered and standard deviation scaled with The Unscrambler (Version 10.0.1, Camo Software Inc., Woodbridge, NJ, USA) for the normalization of the values to give all variables an equal chance. After normalization of all the data sets, normalized values were used in the color scales system from Microsoft Office Excel 2010 (Microsoft Corporation, Redmond, WA, USA) to generate the heatmap.

The normalized data set was used for the principal component analysis (PCA) by performing normalized volume of the differentially abundant protein spots using the Unscrambler (version 10.0.1, Camo Software Inc., Woodbridge, NJ, USA). The PCA loading plots were used to determine the separation of the differentially expressed proteins based on the presence and absence between TPL and MGL samples. The PCA loading plots were performed in triplicate (n = 3).

## Results

### Responses of polyphenolic compounds

**Fig 2:**
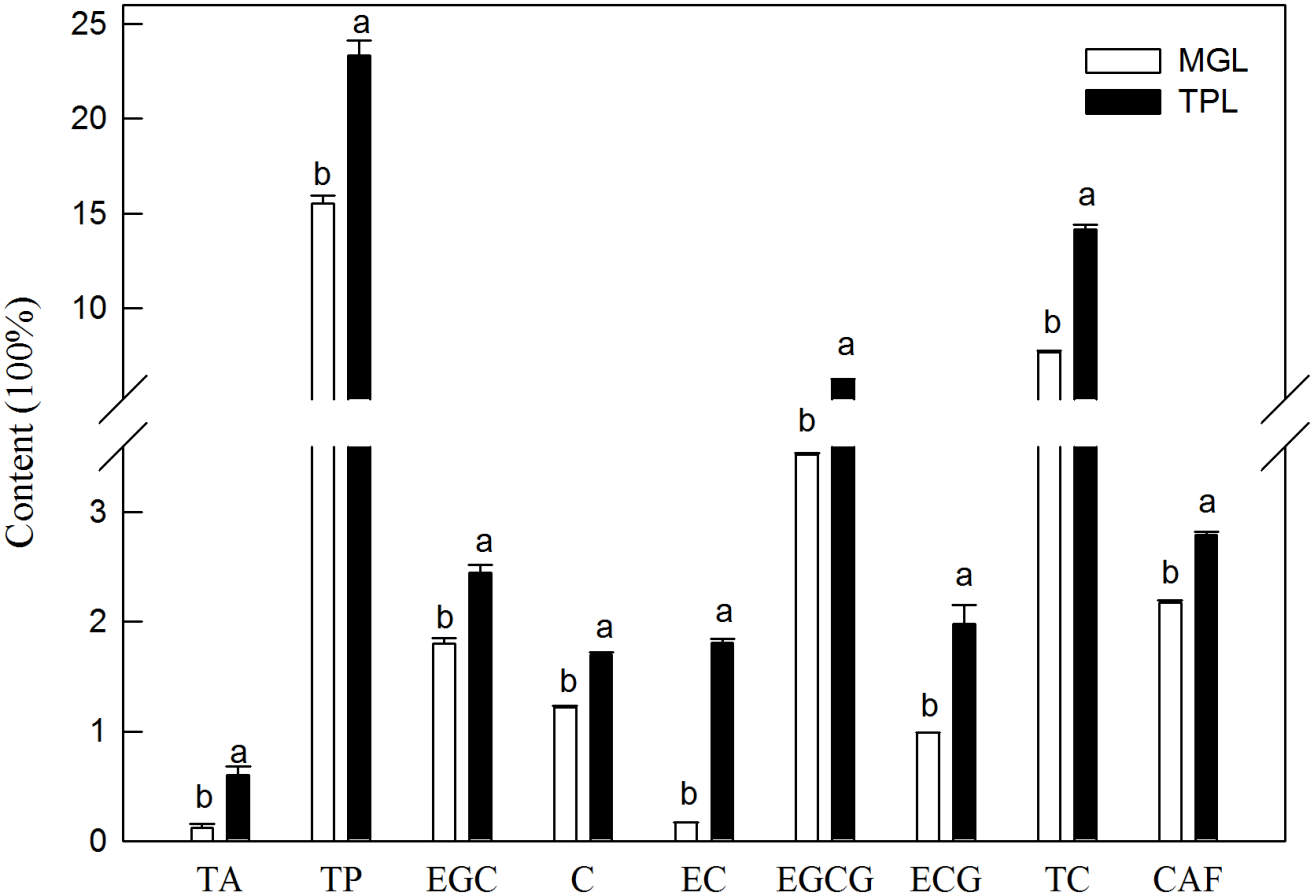
Changes in the contents of total anthocyanins (TA), total polyphenols (TP), (−)-epigallocatechin (EGC), (+)-catechin (C), (−)-epicatechin (EC), (−)-epigallocatechin 3-O-gallate (EGCG), (−)-epicatechin 3-O-gallate (ECG), total catechins (TC) and caffeine (CAF) in mature green leaf (MGL) and tender purple leaf (TPL) tissues. Bars represent means ± SE (n = 4). Different letters above the bars indicate a significant difference at *p*<0.05.

In this study, we analyzed the contents of total anthocyanins (TA), total polyphenols (TP), catechins and caffeine in TPL and MGL tissues. As shown in Fig 2, the concentrations of TA and TP, (−)-epigallocatechin (EGC), (+)-catechin (C), (−)-epicatechin (EC), (−)-epigallocatechin 3-O-gallate (EGCG), (−)-epicatechin 3-O-gallate (ECG), total catechins and caffeine were significantly higher in TPL tissues than in MGL ones. These findings clearly indicate that the content of quality determinants in tea leaves decreases as tea leaves develop.

### Identification of differentially expressed proteins

**Fig 3:**
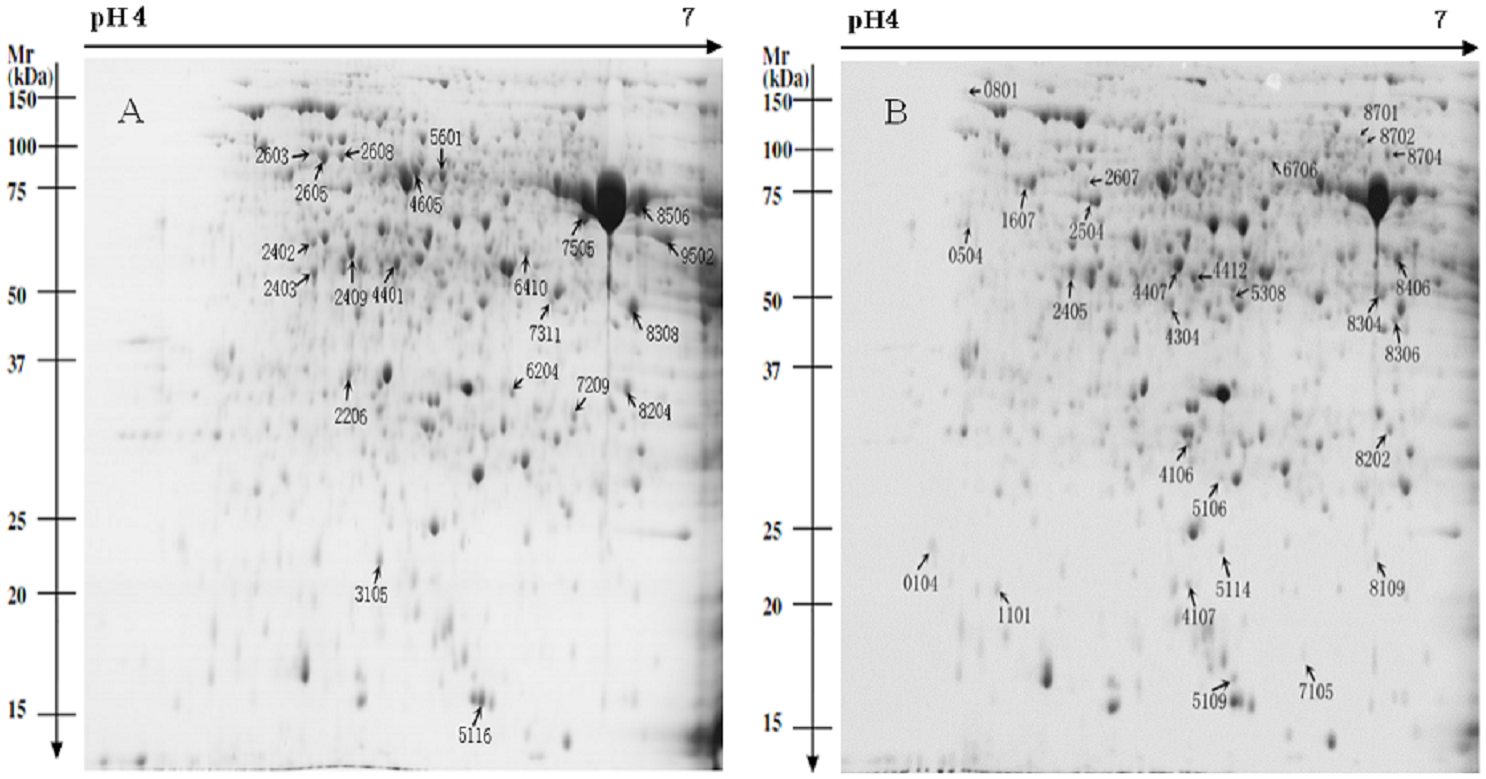
Representative 2-DE gels from *Camellia sinensis* of the development of young shoot purple-related tea plant. **A, mature green leaf (MGL) tissues; B, tender purple leaf (TPL) tissues**. Note: Differentially regulated proteins are numbered and indicated by arrows.

To explore the correlation between the proteomic and metabolite profiles of TPL and MGL tissues, 2-DE coupled with MS/MS technique was carried out with three biological replicates. More than 900 clear and reproducible spots were detected on each gel (Fig 3). After conducting analyses with PDQuest 8.0.1 software and MALDI-TOF/TOF-MS, we successfully identified 46 proteins (S1 Fig, Table 1). Of these identified proteins, 26 proteins had high abundance in TPL tissues, while 20 proteins had low abundance compared with those in MGL tissues. The results from the database are presented in Table 1.

**Table 1:** List of differentially expressed proteins identified using MALDI-TOF/TOF-MS in tender purple leaf (TPL) and mature green leaf (MGL) tissues of *Camellia sinensis*.

Spot No.^a^	Protein	Accession No.^b^	Score	Expect	Species	Mr	Pi	Ratio^c^±SD
***Carbohydrate and energy metabolism***								
2206	Oxygen evolving enhancer protein	gi|326467059	466	6.8e-041	*Litchi chinensis*	39.25	5.35	0.31±0.02
7209	Carbonic anhydrase	gi|427199625	245	8.5e-019	*Vigna unguiculata*	36.54	6.24	0
8304	Cytosolic malate dehydrogenase	gi|301015433	626	6.8e-057	*Camellia sinensis*	48.71	6.4	100
8306	Malate dehydrogenase	gi|114479586	514	1.1e-045	*Citrus junos*	46.03	6.45	100
8308	Fructose-bisphosphate aldolase 3	gi|432139325	414	1.1e-035	*Camellia oleifera*	47.34	6.47	0.49±0.05
4304	Fructokinase	gi|402810391	290	2.7e-023	*Actinidia eriantha*	47.12	5.62	2.27±0.18
2409	Phosphoribulokinase, chloroplastic-like	gi|460400830	262	1.7e-020	*Solanum lycopersicum*	52.31	5.36	0.34±0.02
6410	Phosphoglycerate kinase, chloroplastic-like	gi|356525742	741	2.1e-068	*Glycine max*	53.4	6.06	0.37±0.01
8702	Pyruvate decarboxylase family protein	gi|297319662	151	2.1e-009	*Arabidopsis lyrata subsp*.*lyrata*	69.17	6.33	2.39±0.01
2403	Sedoheptulose-1,7-bisphosphatase, chloroplastic	gi|225466690	423	1.4e-036	*Vitis vinifera*	49.92	5.21	0.37±0.04
7705	Cytochrome b5 DIF-F	gi|4204575	132	1.7e-007	*Petunia x hybrida*	21.13	6.13	100
3105	Cytochrome b6-f complex iron-sulfur subunit	gi|225461287	155	8.50E-10	*Vitis vinifera*	25.52	5.48	0.31±0.01
2608	ATP synthase CF1 alpha subunit (chloroplast)	gi|430728257	984	1.1e-092	*Camellia sinensis*	65.8	5.32	0.44±0.01
2603	ATP synthase CF1 alpha subunit (chloroplast)	gi|430728257	926	6.8e-087	*Camellia sinensis*	66.56	5.2	0.22±0.03
2605	ATP synthase CF1 alpha subunit (chloroplast)	gi|430728257	939	3.4e-088	*Camellia sinensis*	65.7	5.26	0.36±0.02
7311	ATP-dependent clp protease, putative	gi|223550217	395	8.50E-34	*Ricinus communis*	47.61	6.14	0
4401	Ribulose 1,5-bisphosphate carboxylase/oxygenase activase	gi|359478916	320	2.7e-026	*Vitis vinifera*	51.3	5.56	0.11±0.01
2402	Ribulose 1,5-bisphosphate carboxylase/oxygenase activase	gi|158726716	404	1.1e-034	*Flaveria bidentis*	53.85	5.21	0.2±0.05
8204	Ribulose-1,5-bisphosphate carboxylase/oxygenase large subunit	gi|148590322	610	2.7e-055	*Parkia multijuga*	39.21	6.46	0.2±0.02
9502	Ribulose-1,5-bisphosphate carboxylase/oxygenase large subunit	gi|18140571	662	1.7e-060	*Camellia sinensis*	56.55	6.61	0.07±0.02
8506	Ribulose-1,5-bisphosphate carboxylase/oxygenase large subunit	gi|18140563	578	4.30E-52	*Camellia albogigas*	60.29	6.5	0.04±0.01
7505	Ribulose 1,5-bisphosphate carboxylase	gi|17136048	683	1.4e-062	*Tetrameles nudiflora*	58.95	6.27	0.3±0.03
5308	Carbon-nitrogen hydrolase family protein	gi|297317346	88	0.004	*Arabidopsis lyrata*	48.08	5.86	3.33±0.25
***Secondary metabolism***								
4106	Chalcone isomerase	gi|76152009	252	1.7e-019	*Camellia sinensis*	33.98	5.69	2.02±0.17
8406	Chalcone synthase	gi|22086369	128	4.3e-007	*Rubus idaeus*	52.87	6.47	3.13±0.25
2405	Caffeine synthase	gi|145952324	316	6.8e-026	*Camellia sinensis*	51.14	5.26	2.53±0.1
4412	Flavonol synthase	gi|76786311	705	8.5e-065	*Camellia sinensis*	50.17	5.71	2.43±0.19
***Protein metabolism***								
5601	Anthranilate N-benzoyltransferase protein, putative	gi|223547579	105	8.5e-005	*Ricinus communis*	63.79	5.74	0.33±0.03
8701	TCP-1/cpn60 chaperonin family protein	gi|332006171	205	8.5e-015	*Arabidopsis thaliana*	69.81	6.31	2.43±0.1
4407	Glutamine synthetase	gi|42733460	570	2.70E-51	*Camellia sinensis*	51.49	5.65	2.9±0.07
6706	T-complex protein 1 subunit beta	gi|225459806	440	2.7e-038	*Vitis vinifera*	66.93	6	3.08±0.25
8202	proteasome subunit alpha type-6 isoform	gi|359482460	334	1.1e-027	*Vitis vinifera*	35.66	6.44	2.02±0.14
2607	alanine aminotransferase 2, mitochondrial-like, partial	gi|449519802	150	2.7e-009	*Cucumis sativus*	63.26	5.31	100
801	Protein disulfide isomerase-like 1-4-like isoform 2	gi|356539444	81	0.02	*Glycine max*	76.99	4.85	2.22±0.64
***Nucleic acid metabolism***								
5109	Glycine-rich RNA-binding protein	gi|187373099	323	1.4e-026	*Nicotiana tabacum*	19.97	5.86	100
8704	Bifunctional purine biosynthesis protein, putative	gi|223529724	162	1.70E-10	*Ricinus communis*	67.22	6.43	5±0.48
***Cell wall*, *cytoskeleton and transport***								
2504	alpha-tubulin	gi|385717688	892	1.7e-083	*Oryza sativa Japonica Group*	60.71	5.32	100
1607	Tubulin, beta chain	gi|222872170	721	2.1e-066	*Populus trichocarpa*	62.8	5.09	2.39±0.28
504	Bark storage protein A	gi|225429975	116	6.8e-006	*Vitis vinifera*	56.4	4.85	2.24±0.21
***Stress responses***								
5116	Copper/zinc superoxide dismutase 1	gi|381283808	373	1.4e-031	*Litchi chinensis*	18.98	5.9	0.48±0.04
6204	Thioredoxin-like protein CDSP32, chloroplastic	gi|225459760	324	1.1e-026	*Vitis vinifera*	38.33	6	0.46±0.03
4107	Cold shock domain protein	gi|187609563	152	1.7e-009	*Oryza sativa Japonica Group*	24.74	5.69	2.72±0.04
5106	Glutathione S-transferase	gi|76365795	124	1.1e-006	*Citrus sinensis*	31.77	5.82	100
5114	Phospholipid hydroperoxide glutathione peroxidase	gi|34786892	400	2.7e-034	*Citrus sinensis*	27	5.82	2.05±0.07
104	Translationally controlled tumor protein	gi|158120965	168	4.3e-011	*Salvia miltiorrhiza*	27.22	4.71	3.58±0.74
***Other and unknown biological processes***								
8109	predicted protein	gi|222857852	63	1.1e-005	*Populus trichocarpa*	26.83	6.39	100

^a^: Spot number corresponds to the 2-DE gel in Fig 3.

^b^: gi number is from NCBI database of matched protein.

^c^: Ratio means the ratio of TPL to MGL; 0 means protein spots were only detected in MGL tissues; 100 means protein spots were only detected in the TPL tissues.

**Fig 4:**
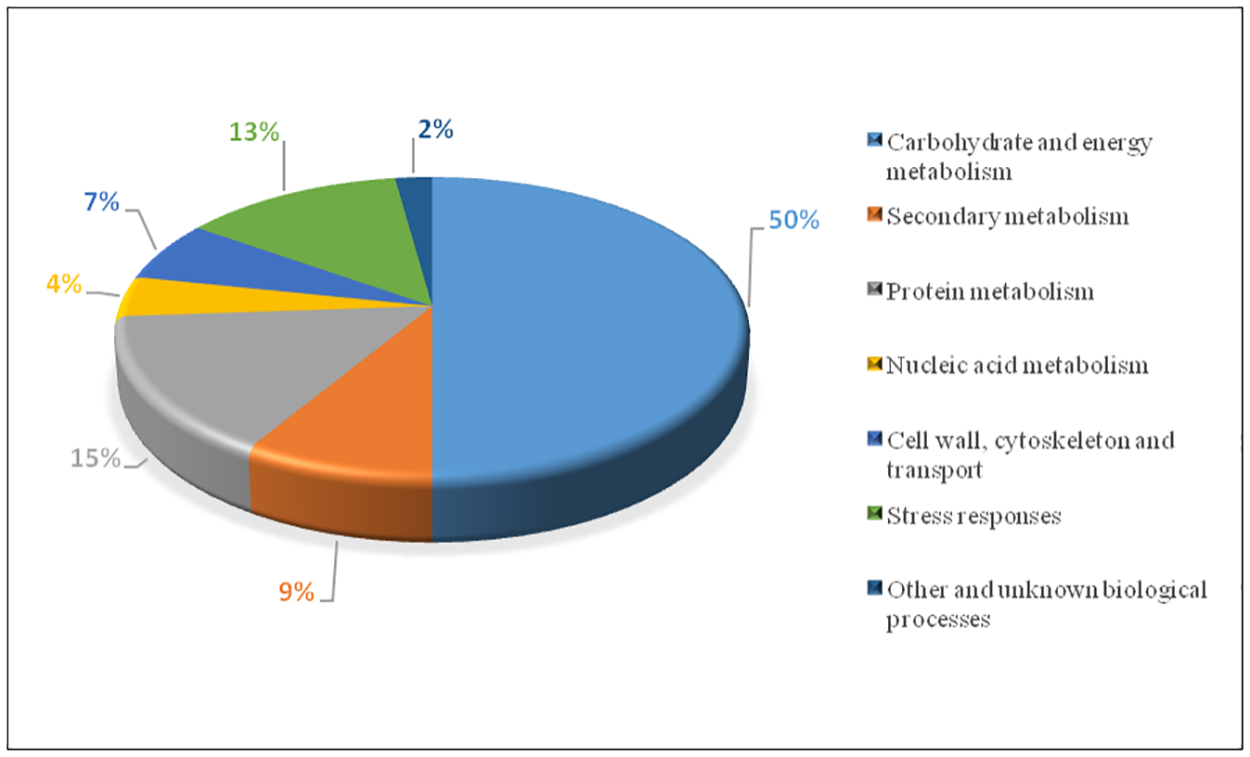
Functional classification of differentially expressed transcript derived fragments.

According to biological functional properties, the 46 differentially abundant proteins were categorized into the following functional groups: carbohydrate and energy metabolism (50%), protein metabolism (15%), secondary metabolism (9%), stress responses (13%), nucleic acid metabolism (4%), cell wall, cytoskeleton and cell transport (7%), other and unknown biological processes (2%) (Fig 4).

### PCA and heatmap responses

**Fig 5:**
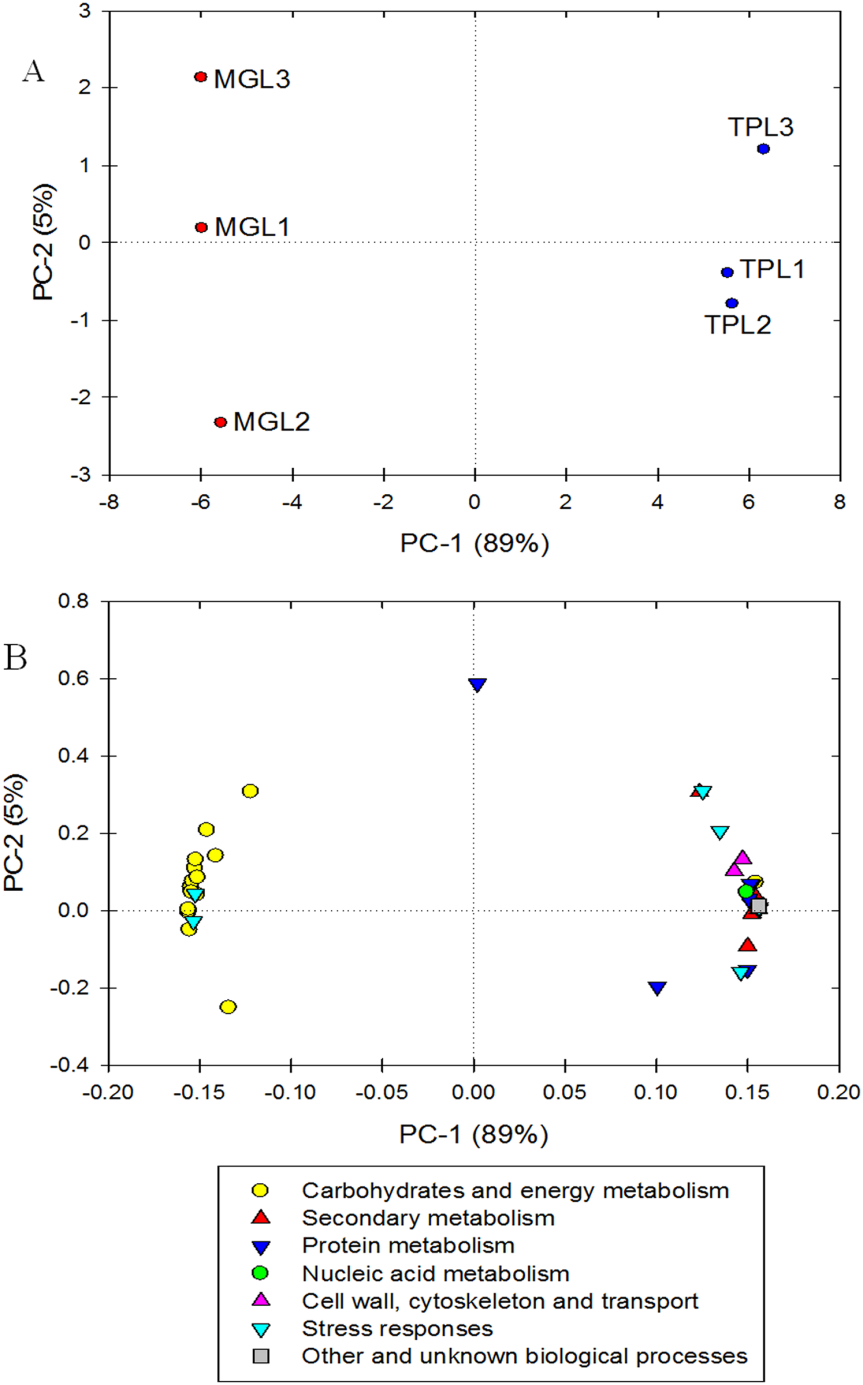
Principal component analysis (PCA) of scores (A) and loading (B) plots of proteomic responses from mature green leaf (MGL) and tender purple leaf (TPL) tissues of tea plants.

**Fig 6:**
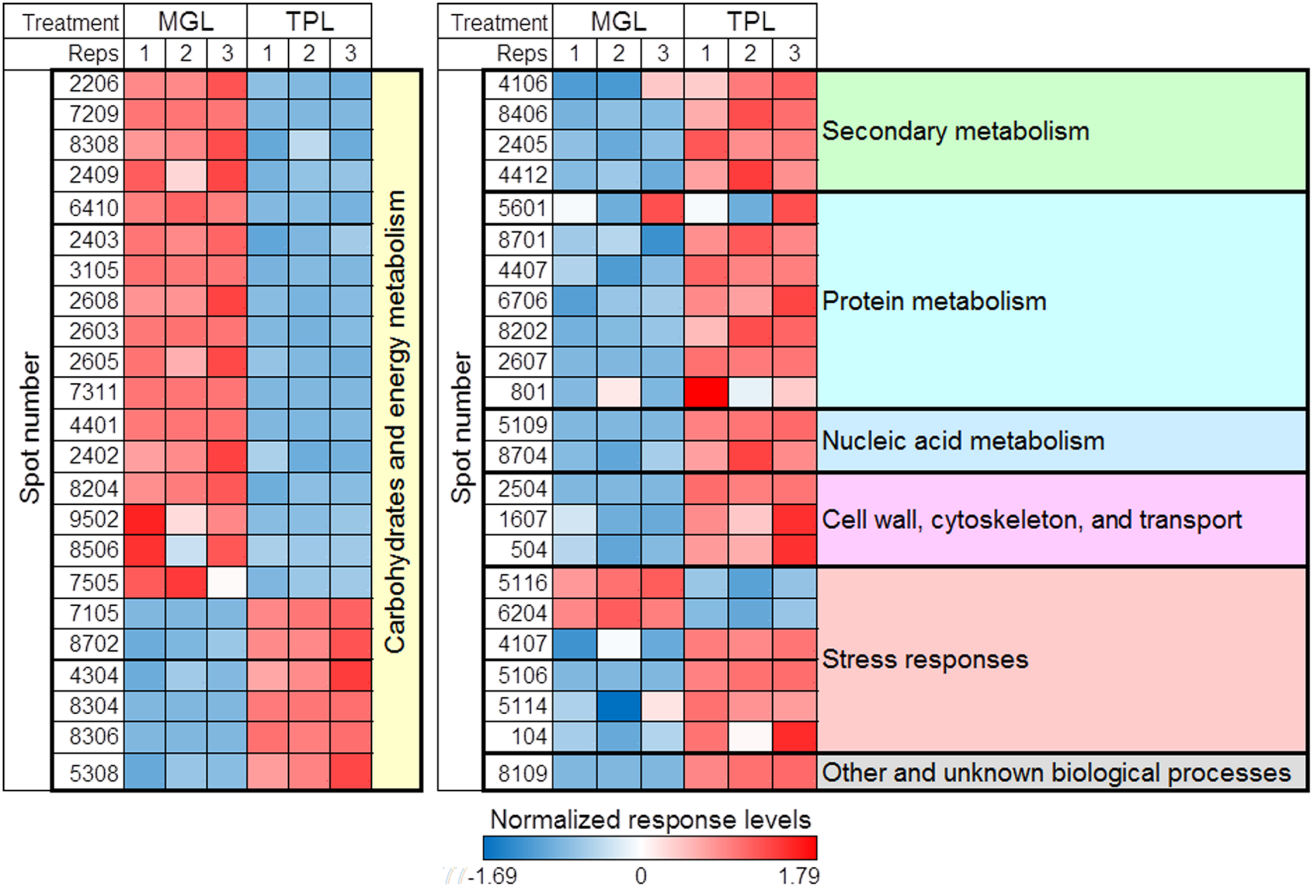
Heatmap of proteomic responses from mature green leaf (MGL) and tender purple leaf (TPL) tissues of tea plants. Proteomic levels correspond to the color scale. A color gradient from blue through to red represents a low level through to a high level of proteomic response. After calculating the mean of each metabolite, the normalized data were generated using the Unscrambler software.

PCA was performed with all 46 of the identified proteins. The PC1 and PC2 values, which accounted for 89% and 5% of the total X and Y variance, revealed that the differential protein responses between TPL and MGL were clearly separated by the first principal component (Fig 5A). Interestingly, the majority of differentially expressed proteins involved in carbohydrate and energy metabolism were highly clustered (Fig 5B). The heatmap of protein responses in TPL and MGL samples shows the correlation patterns of the differentially abundant proteins (Fig 6).

### Transcriptional expression analysis of differentially abundant proteins

**Fig 7:**
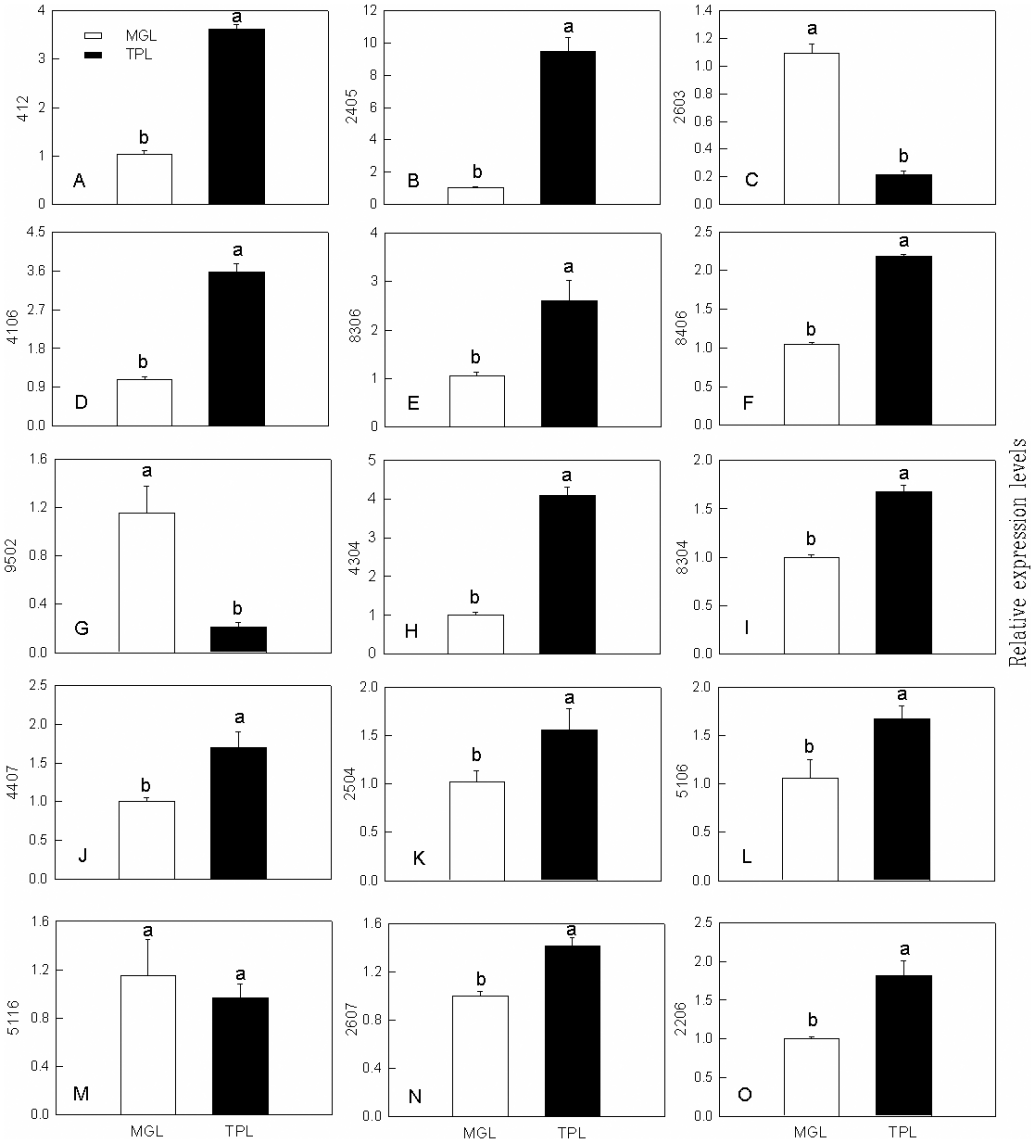
Relative expression levels of genes encoding (A) flavonol synthase (S412), (B) caffeine synthase (S2405), (C) ATP synthase CF1 alpha subunit (S2603), (D) chalcone isomerase (S4106), (E) malate dehydrogenase (S8306), (F) chalcone synthase (S8406), (G) ribulose-1,5-bisphosphate carboxylase (S9502), (H) fructokinase, (I) cytosolic malate dehydrogenase, (J)glutamine synthetase, (K) alpha-tubulin, (L) glutathione *S*-transferase, (M) copper/zinc superoxide dismutase, (N) alanine aminotransferase, (O) oxygen evolving enhancer protein from *Camellia sinensis* leaves revealed by qPCR. Bars represent means ± SE (n = 9). Different letters above standard error bars indicate significant differences at *p* < 0.05.

To verify the proteomic results and evaluate the association between mRNA and protein levels, qPCR assays were conducted (Fig 7). From all identified proteins, fifteen gene products were selected to analyze the expression patterns of their mRNA levels: flavonol synthase (spot No. 412), chalcone synthase (spot No. 8406), ATP synthase CF1 alpha subunit (spot No. 2603), chalcone isomerase (spot No. 4106), malate dehydrogenase (spot No. 8306), chalcone synthase (spot No. 8406) and rubisco (spot No. 9502), fructokinase (spot No. 4304), cytosolic malate dehydrogenase (spot No. 8304), glutamine synthetase (spot No. 4407), alpha-tubulin (spot No. 2504), glutathione *S*-transferase (spot No. 5106), copper/zinc superoxide dismutase (spot No. 5116), alanine aminotransferase (spot No. 2607) and oxygen evolving enhancer protein (spot No. 2206). The expression levels of genes encoding flavonol synthase (Fig 7A), caffeine synthase (Fig 7B), chalcone isomerase (Fig 7D), malate dehydrogenase (Fig 7E), chalcone synthase (Fig 7F), fructokinase (Fig 7H), cytosolic malate dehydrogenase (Fig 7I), glutamine synthetase (Fig 7J), alpha-tubulin (Fig 7K), glutathione *S*-transferase (Fig 7L) and alanine aminotransferase (Fig 7N) were higher in TPL tissues than in MGL ones, while the expression levels of genes encoding the ATP synthase CF1 alpha subunit (Fig 7C), rubisco (Fig 7G), copper/zinc superoxide dismutase (Fig 7M) and oxygen evolving enhancer protein (Fig 7O) were lower in TPL tissues than in MGL ones. As shown in Fig 7, the transcription profiles of these genes matched the protein expression levels well, indicating that the levels of differentially abundant proteins were regulated at the transcriptional level. Only one gene encoding oxygen evolving enhancer protein exhibited different expression patterns under qPCR analysis compared with proteomic evaluation. The different results may be attributed to the posttranscriptional regulatory process and the different sensitivities of the analytical methods.

## Discussion

Anthocyanins often accumulate in young leaves, fruit or flowers, and then degrade as leaves develop [28]. This tendency has been reported on many plant species, including *Chrysobalanus icaco* [29], *Capsicum* spp [30] and *Brunfelsia calycina* [31*]*. A previous study by our group also showed that anthocyanin contents decreased with leaf growth and development, while the contents of chlorophyll *a* and chlorophyll *b* were higher in MGL samples. Thus, we used the cDNA-AFLP technique to reveal the regulatory mechanism of the alterations of leaf color during leaf development in Wuyiqizhong18 [19]. Nevertheless, due to post-transcriptional regulation, gene transcription data are insufficient to predict the expression levels of proteins. To further understanding the molecular mechanism underling the development of tea leaves, we used 2-DE technique to investigate the proteomic profile of different leaves and successfully identified 46 differentially abundant proteins in MGL and TPL samples in this study.

### Proteins involved in carbohydrate and energy metabolism

**Fig 8:**
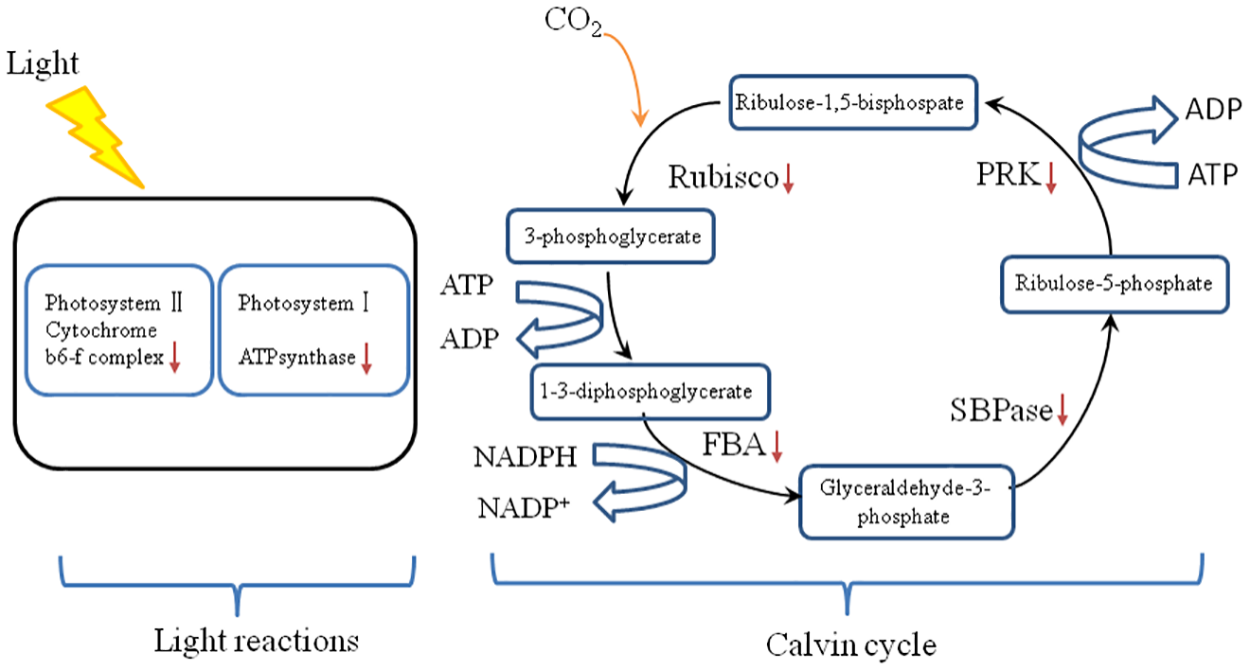
The differentially abundant proteins in the light reactions and Calvin cycle in tender leaves of *Camellia sinensis*. Note: Rubisco: Ribulose-1,5-bisphosphate carboxylase/oxygenase; FBA: Fructose-bisphosphate aldolase; SBPase: Sedoheptulose-1,7-bisphosphatase; PRK: Phosphoribulokinase; Arrows represent meansLow abundance.

We identified 6 increased and 17 decreased proteins that were involved in carbohydrate and energy metabolisms from TPL tissues (Table 1). In plants, the tricarboxylic acid cycle (TCA cycle) and the pentose phosphate pathway (PPP) provide not only energy and cofactors but also substrates for the synthesis of metabolites, or signals for feedback [32]. Interestingly, in the present study, we found that some key enzymes involved in carbon assimilation had high abundance in TPL, such as phosphoglycerate kinase (PGK, spot No. 6410), fructose-bisphosphate aldolase (spot No. 8308), sedoheptulose-1,7-bisphosphatase (spot No. 2403) and phosphoribulokinase (PRK, spot No. 2409) (Fig 8). Moreover, TPL sample has a lower abundance of rubisco (spot No. 4401, 2402, 8204, 9502, 8506 and 7505), a bi-functional enzyme that catalyzes the initial step of the Calvin cycle and the initial oxygenation reaction of photorespiration. This observation was well corresponded with our previous study, reporting that the transcriptional levels of the genes encoding some photosynthetic enzymes are decreased in TPL tissues [19]. The lower abundance of rubiscos would be responsible for the lower activity of CO_2_ assimilation in TPL tissues than in MGL ones. It is also possible that the lower abundance of rubiscos in TPL tissues due to the immature chloroplasts of TPL tissues [18].

Four enzymes (malate dehydrogenase, spot No. 8304 and 806; fructokinase, spot No. 4304; pyruvate decarboxylase, spot No. 8702) involved in organic acid metabolism and glycolysis have significantly higher abundance in TPL tissues than in MGL ones. It means that energy and secondary metabolism would be enhanced in TPL tissues. Previous research has suggested that anthocyanins in young leaves are likely associated with tolerance against photoinhibition by attenuating visible radiation [33, 34]. Since TPL tissues are rich in anthocyanins, light attenuation by anthocyanins may display shade acclimation of the photosynthetic machinery in purple leaves, which is compatible with a photoprotective function of anthocyanins [35]. The accumulation of anthocyanins in TPL tissues may serve as a filter of light and protect chloroplasts against photoinhibition [36]. Thus, anthocyanins likely play a key role in the photoprotection of young leaves, which is a hypothesis that has been addressed in a previous study [37].

### Proteins involved in secondary metabolism

Although the proportion of differentially abundance proteins related to secondary metabolism in our results was relatively low (9%) compared with the other categories, proteins related to anthocyanin and caffeine biosynthesis were found to be differentially abundances between TPL and MGL tissues (Table 1). Tea leaves are a valuable source of secondary metabolites, including polyphenols, alkaloids, volatile oils, and others [2, 38]. Based on the proteomic results in this study, several proteins related to polyphenol biosynthesis were differentially expressed between the TPL and MGL tissues. CHS is the first committed enzyme involved in anthocyanin and flavonoid biosynthesis [39]. Gene silencing (via RNA interference or virus-induced gene silencing) of CHS in both of *Torenia hybrida* and petunia resulted in lower contents of total flavonoid and anthocyanins, and CHS enzyme activity of flower tissues [39, 40]. In the present study, the abundance of CHS (spot No. 8406) was increased in TPL tissues, indicating that the biosysnthesis of anthocyanin and flavonoid was enhanced. This outcome was consistent with our previous finding that CHS expression was a positive regulator of anthocyanin biosynthesis [19]. Furthermore, qPCR was employed to verify the higher gene expression level of CHS in TPL tissues (Fig 7F). CHI and FLS are two key enzymes involved in the flavonoid biosynthesis pathway [41–42]. In CHI-suppressed transgenic tobacco plants, they showed up to 25% reduction in anthocyanins content [43]. In the present study, the abundance of CHI (spot No. 4106) and FLS (spot No. 412) were higher in TPL tissues, which was consistent with the qPCR results, than in MGL ones (Fig 7). Thus, higher abundance of CHI, CHS and FLS may account for the production of the purple-shoot phenotype in Wuyiqizhong 18 and enhance the flavonoid biosynthesis. These results were also agreed with our metabolic data, which show that the contents of TA and TP were significantly higher in TPL tissues than in MGL ones (Fig 2).

### Proteins involved in stress responses

The production of reactive oxidative species (ROS) is inevitable in plant grown in aerobic circumstance. As an adaptive strategy, plants have evolved several capabilities to scavenge ROS including endogenous antioxidant enzymes system and antioxidant products, such as superoxide dismutase (SOD), catalase (CAT), glutathione peroxidase (GPx), and glutathione reductase (GR). Due to different sub-cellular localization, the abundance of these anti-oxidative enzymes did not change in the same pattern in response to an environmental stimulus [44]. In our study, four proteins involved in stress responses have higher abundance in TPL tissues, while one protein named copper/zinc superoxide dismutase (Cu/Zn SOD, spot No. 5116) has lower abundance in TPL tissues than in MGL ones (Table 1; Fig 7M). Cu/Zn SOD catalyzes the dismutation of ion superoxide into oxygen and hydrogen peroxide in plant chloroplast [45]. The lower abundance of Cu/Zn SOD in TPL tissues might be due to the well protection of anthocyanins. PHGPx, one of the protein isoforms of GPx, protects plants from pathogen attack and tolerance to other abiotic stresses [46]. A previous study suggested that only part of the absorbed light energy is utilized in photosynthesis, and the excess photon flux could lead to photo-damage and ROS production due to decreased CO_2_ assimilation in TPL tissues [19, 47]. As hypothesis, the increased abundance of PHGPx (spot No. 5114) and GST (spot No. 5106) observed in TPL tissues could quench the generated ROS by excess light and ameliorate the oxidative damage caused by ROS. Moreover, GPx and GSTs with GPx activity also decompose other toxic compound like alkyl hydroperoxides in addition to H_2_O_2_ generated by SODs activity [48]. Based on these results, we concluded that the accumulation of stress-related proteins in TPL tissues might dissipate the excess excitation energy and protect tender leaves against photo-damage.

### Proteins involved in protein metabolism

Most of the proteins involved in protein metabolism have higher abundance in TPL tissues, compared with those in MGL tissues. In this study, the higher abundance of protein disulfide isomerase-like 1-4-like isoform 2 (PDIL, spot No.801), glutamine synthetase (GS, spot No.4407) and chaperonin family proteins (spot No.8701) indicated that there was an increased requirement for protein synthesis, correct assembly and turnover due to the development of tender leaves. Especially, GS (EC 6.3.1.2) is a key enzyme involved in the assimilation of ammonium into glutamine (Gln) [49, 50]. GS plays an important role in the synthesis of theanine, which is a unique amino acid found in tea plants and is the main component responsible for the taste of tea [51]. GS showed higher in tender leaves and lower abundance in older leaves, indicating that the synthesis of theanine in tender leaves was enhanced [19, 52].

### Proteins involved in nucleic acid metabolism, the cellular cytoskeleton and transport

Glycine-rich RNA-binding proteins (GRPs) harbor an RNA-recognition motif (RRMs) at the N-terminus and a glycine-rich region at the C-terminus [53]. The expression patterns of GRPs are regulated by a number of external stimuli, such as cold stress conditions [54], high salinity and osmotic stress [55]. The high abundances of GRPs (spot No.5109) in TPL tissues could enhance stress resistance of tender tissue. Microtubules are dynamic heteropolymers of α- and β-tubulin, which are structural component of the cytoskeleton in eukaryote [56]. It plays key roles in many basic processes of eukaryotic cells, such as cell division, cell motility, intracellular transport and the control of cell shape [57]. The high abundance of α-tubulin (spot No. 2504) and β-tubulin (spot No. 1607) could maintain the stability of the cytoskeleton system, which is very important for the development of plants.

## Conclusions

Tea plants with purple coloration represent important tea germplasms due to their economic value and health benefits. The pronounced changes in the proteomic profile between TPL and MGL tissues were included: 1) the lower activity of proteins associated with CO_2_ assimilation, energy metabolism and photo flux efficiency and higher content of anthocyanins in TPL samples may protect tender leaves against photo-damage; 2) the higher abundance of CHS, CHI and FLS likely contributes to the synthesis of anthocyanins, catechins and flavonols in TPL tissues; 3) higher abundance of stress response proteins, such as GST and PHGPx, could enhance the tolerance of TPL tissues to adverse condition; and 4) the increased abundance of proteins related to protein synthesis, nucleic acids and cell wall proteins should be beneficial for the proliferation and expansion of leaf cell in TPL tissues. In summary, the results revealed that the higher abundance of two key proteins including CHS and CHI were responsible for the higher content of anthocyanins in TPL tissues compared with MGL ones. The higher abundance of GS protein related to the theanine biosynthesis may improve the flavor of tea products from TPL materials. This work enhances our understanding of the molecular mechanisms behind the changes in leaf color alteration between TPL and MGL tissues. Therefore, future studies should focus on the regulatory role of CHS and CHI in anthocyanin biosynthesis pathway of purple leaves of tea plants.

## Supporting information

S1 Fig Total ion chromatogram of protein spot 8406 excised from a 2-DE gel, trypsin-digested and analyzed by MALDI-TOF/TOF-MS. (A) Peptide mass fingerprinting of protein spot 8406; (B) MS/MS spectra of ion 1799.8, the corresponding peptide sequence are shown.
(TIF)

S2 Fig Replicated gels of mature green leaf (MGL) and tender purple leaf (TPL) tissues. (DOCX)

S3 Fig Master gel. (PDF)

S1 Table Primer sequences used for quantitative real-time PCR. (DOCX)

## References

[1] Sharma RK, Bhardwaj PB, Negi R, Mohapatra T, Ahuja PS. Identification, characterization and utilization of unigene derived microsatellite markers in tea (*Camellia sinensis* L.). BMC Plant Biol. 2009; 9: 1–24. 19426565 10.1186/1471-2229-9-53 PMC2693106

[2] Yamamoto T, Juneja LR, Kim M. Chemistry and applications of green tea. CRC press, New York 1997, 160.

[3] Hodgson JM, Croft KD. Tea flavonoids and cardiovascular health. Mol. Aspects Med. 2010; 31: 495–502. 10.1016/j.mam.2010.09.004 20837049

[4] Geleijnse JM, Launer LJ, Hofman A, Pols HA, Witteman JC. Tea flavonoids may protect against atherosclerosis: the Rotterdam Study. Arch. Inter. Med. 1999; 159: 2170–2174. 10.1001/archinte.159.18.2170 10527294

[5] Higdon JV, Frei B. Tea catechins and polyphenols: health effects, metabolism, and antioxidant functions. Crit. Rev. Food Sci. 2003; 43: 89–143. 10.1080/10408690390826464 12587987

[6] Fujiki H. Green tea: Health benefits as cancer preventive for humans. Chem. Rec. 2005; 5: 119–132. 10.1002/tcr.20039 15889414

[7] Joshi R, Rana A, Gulati A. Studies on quality of orthodox teas made from anthocyanin-rich tea clones growing in Kangra valley, India. Food Chem. 2015; 176: 357–366. 10.1016/j.foodchem.2014.12.067 25624244

[8] Hsu CP, Shih YT, Lin BR, Chiu CF, Lin CC. Inhibitory effect and mechanisms of an anthocyanins- and anthocyanidins-rich extract from purple-shoot tea on colorectal carcinoma cell proliferation. J. Agric. Food Chem. 2012; 60: 3686–3692. 10.1021/jf204619n 22404116

[9] Rashid K, Wachira FN, Nyabuga JN, Wanyonyi B, Murilla G, Isaac AO. Kenyan purple tea anthocyanins ability to cross the blood brain barrier and reinforce brain antioxidant capacity in mice. Nutr. Neurosci. 2014; 17(4): 178–185. 10.1179/1476830513Y.0000000081 23883519

[10] Zafra-Stone S, Yasmin T, Bagchi M, Chatterjee A, Vinson JA, Bagchi D. Berry anthocyanins as novel antioxidants in human health and disease prevention. Mol. Nutr. Food Res. 2007; 51: 675–683. 10.1002/mnfr.200700002 17533652

[11] Kerio L, Wachira F, Wanyoko J, Rotich M. Characterization of anthocyanins in Kenyan teas: extraction and identification. Food Chem. 2012; 131: 31–38.

[12] Jiang L, Shen X, Shoji T, Kanda T, Zhou J, Zhao L. Characterization and activity of anthocyanins in Zijuan Tea (*Camellia sinensis* var. kitamura). J. Agric. Food Chem. 2013; 61: 3306–3310. 10.1021/jf304860u 23477682

[13] Nesumi A, Ogino A, Yoshida K, Taniguchi F, Maeda YM, Tanaka J, Murakami A. ‘Sunrouge’, a new tea cultivar with high anthocyanin. JARQ-Jpn. Agr. Res. Q. 2012; 46: 321–328.

[14] Joshi R, Rana A, Gulati A. Studies on quality of orthodox teas made from anthocyanin-rich tea clones growing in Kangra valley, India. Food Chem. 2015; 176: 357–366. 10.1016/j.foodchem.2014.12.067 25624244

[15] Kilel E, Faraj A, Wanyoko J, Wachira F, Mwingirwa V. Green tea from purple leaf coloured tea clones in Kenya-their quality characteristics. Food Chem. 2013; 141: 769–775. 10.1016/j.foodchem.2013.03.051 23790846

[16] Kerio L, Wachira F, Wanyoko J, Rotich M. Total polyphenols, catechin profiles and antioxidant activity of tea products from purple leaf coloured tea cultivars. Food Chem. 2013; 136:1405–1413. 10.1016/j.foodchem.2012.09.066 23194541

[17] fgcChalker-Scott L. Environmental significance of anthocyanins in plant stress responses. Photochem. Photobiol. 1999; 70: 1–9.

[18] Maayan I, Shaya F, Ratner K, Mani Y, Lavee S, Avidan B, et al Photosynthetic activity during olive (*Olea europaea*) leaf development correlates with plastid biogenesis and Rubisco levels. Physiol. Plant. 2008; 134: 547–558. 10.1111/j.1399-3054.2008.01150.x 18636989

[19] Zhou QQ, Sun WJ, Lai ZX. Differential expression of genes in purple-shoot tea tender leaves and mature leaves during leaf growth. J. Sci. Food Agric. 2016; 96: 1982–1989. 10.1002/jsfa.7308 26084622

[20] Hatlestad GJ, Akhavan NA, Sunnadeniya RM, Elam L, Cargile S, Hembd A, et al The beet Y locus encodes an anthocyanin MYB-like protein that activates the betalain red pigment pathway. Nat. Genet. 2015; 47: 92–96. 10.1038/ng.3163 25436858

[21] Wei K, Wang LY, Zhou J, He W, Zeng JM, Jiang YW, Cheng H. Comparison of catechins and purine alkaloids in albino and normal green tea cultivars (*Camellia sinensis* L.) by HPLC. Food Chem. 2012; 130: 720–724.

[22] Saravanan RS, Rose JK. A critical evaluation of sample extraction techniques for enhanced proteomics analysis of recalcitrant plant tissues. Proteomics. 2004; 4: 2522–2532. 10.1002/pmic.200300789 15352226

[23] Bradford MM. A rapid and sensitive method for quantitation of microgram and quantities of protein utilizing the principle of protein-dye binding. Anal. Biochem. 1976; 72: 248–254. 942051 10.1016/0003-2697(76)90527-3

[24] You X, Yang LT, Lu YB, Li H, Zhang SQ, Chen LS. Proteomic changes of Citrus roots in response to long-term manganese toxicity. Trees. 2014; 28: 1383–1399.

[25] Katam R, Sakata K, Suravajhala P, Pechan T, Kambiranda DM, Naik KS, et al Comparative leaf proteomics of drought-tolerant and-susceptible peanut in response to water stress. J Proteomics. 2016; 143: 209–226. 10.1016/j.jprot.2016.05.031 27282920

[26] Tatusov RL, Galperin MY, Natale DA, Koonin EV, The COG database: a tool for genome-scale analysis of protein functions and evolution. Nucleic Acids Res. 2000; 28: 33–36. 10592175 10.1093/nar/28.1.33 PMC102395

[27] Livak KJ, Schmittgen TD. Analysis of relative gene expression data using real-time quantitative PCR and the 2^− ΔΔCT^ method. Methods. 2001; 25: 402–408. 10.1006/meth.2001.1262 11846609

[28] Oren-Shamir M. Does anthocyanin degradation play a significant role in determining pigment concentration in plants? Plant Sci. 2009; 177: 310–316.

[29] Nissim-Levi A, Kagan S, Ovadia R, Oren-Shamir M. Effects of temperature, UV-light and magnesium on anthocyanin pigmentation in cocoplum leaves. J. Hortic. Sci. Biotech. 2003; 78: 61–64.

[30] Borovsky Y, Oren-Shamir M, Ovadia R, De JW, Paran I. The A locus that controls anthocyanin accumulation in pepper encodes a MYB transcription factor homologous to Anthocyanin 2 of Petunia. Theor. Appl. Genet. 2004; 109: 23–29. 10.1007/s00122-004-1625-9 14997303

[31] Vaknin H, Bar-Akiva A, Ovadia R, Nissim-Levi A, Forer I, Weiss D, et al Active anthocyanin degradation in *Brunfelsia calycina* (yesterday—today—tomorrow) flowers. Planta. 2005; 222: 19–26. 10.1007/s00425-005-1509-5 15918029

[32] Caruso G, Cavaliere C, Guarino C, Gubbiotti R, Foglia P, Laganà A. Identification of changes in Triticum durum L. leaf proteome in response to salt stress by two-dimensional electrophoresis and MALDI-TOF mass spectrometry. Anal. Bioanal. Chem. 2008; 391: 381–390. 10.1007/s00216-008-2008-x 18365183

[33] Manetas Y, Drinia A, Petropoulou Y. High contents of anthocyanins in young leaves are correlated with low pools of xanthophyll cycle components and low risk of photoinhibition. Photosynthetica. 2002; 40: 349–354.

[34] Zhang K, Wang X, Cui J, Ogweno J, Shi K, Zhou Y, et al Characteristics of gas exchange and chlorophyll fluorescence in red and green leaves of Begonia semperflorens. Biol. Plant. 2011; 55: 361–364.

[35] Manetas Y, Petropoulou Y, Psaras GK, Drinia A. Exposed red (anthocyanic) leaves of Quercus coccifera display shade characteristics. Funct. Plant Biol. 2003; 30: 265–270. 10.1071/FP02226 32689008

[36] Terashima I, Fujita T, Inoue T, Chow WS, Oguchi R. Green light drives leaf photosynthesis more efficiently than red light in strong white light: revisiting the enigmatic question of why leaves are green. Plant Cell Physiol. 2009; 50: 684–697. 10.1093/pcp/pcp034 19246458

[37] Steyn W, Wand S, Holcroft D, Jacobs G. Anthocyanins in vegetative tissues: a proposed unified function in photoprotection. New Phytol. 2002; 155: 349–361. 10.1046/j.1469-8137.2002.00482.x 33873306

[38] Li CF, Zhu Y, Yu Y, Zhao QY, Wang SJ, Wang XC, et al Global transcriptome and gene regulation network for secondary metabolite biosynthesis of tea plant (*Camellia sinensis*). BMC genomics. 2015; 16(1): 560. 26220550 10.1186/s12864-015-1773-0 PMC4518527

[39] Fukusaki EI, Kawasaki K, Kajiyama SI, An CI, Suzuki K, Tanaka Y, et al Flower color modulations of Torenia hybrida by downregulation of chalcone synthase genes with RNA interference. J. Biotechnol. 2004; 111: 229–240. 10.1016/j.jbiotec.2004.02.019 15246659

[40] Chen JC, Jiang CZ, Gookin T, Hunter D, Clark D, Reid M. Chalcone synthase as a reporter in virus-induced gene silencing studies of flower senescence. Plant Mol. Biol. 2004; 55: 521–530. 10.1007/s11103-004-0590-7 15604697

[41] Li F, Jin Z, Qu W, Zhao D, Ma F. Cloning of a cDNA encoding the Saussurea medusa chalcone isomerase and its expression in transgenic tobacco. Plant Physiol. Biochem. 2006; 44: 455–461. 10.1016/j.plaphy.2006.08.006 17010632

[42] Muir SR, Collins GJ, Robinson S, Hughes S, Bovy A, De VC, et al Overexpression of petunia chalcone isomerase in tomato results in fruit containing increased levels of flavonols. Nat. Biotechnol. 2001; 19: 470–474. 10.1038/88150 11329019

[43] Nishihara M, Nakatsuka T, Yamamura S. Flavonoid components and flower color change in transgenic tobacco plants by suppression of chalcone isomerase gene. FEBS Lett. 2005; 579: 6074–6078. 10.1016/j.febslet.2005.09.073 16226261

[44] Zhai CZ, Zhao L, Yin LJ, Chen M, Wang QY, Li LC, et al Two wheat glutathione peroxidase genes whose products are located in chloroplasts improve salt and H_2_O_2_ tolerances in Arabidopsis. PLoS One. 2013; 8(10): e73989 10.1371/journal.pone.0073989 24098330 PMC3788784

[45] Molina-Rueda JJ, Tsai CJ, Kirby EG. The Populus superoxide dismutase gene family and its responses to drought stress in transgenic poplar overexpressing a pine cytosolic glutamine synthetase (GS1a). PLoS One. 2013; 8(2): e56421 10.1371/journal.pone.0056421 23451045 PMC3579828

[46] Li WJ, Feng H, Fan JH, Zhang RQ, Zhao NM, Liu JY. Molecular cloning and expression of a phospholipid hydroperoxide glutathione peroxidase homolog in *Oryza sativa*. BBA-Gene Struct. Expr. 2000; 1493: 225–230. 10.1016/s0167-4781(00)00152-4 10978528

[47] Peng HY, Qi YP, Lee J, Yang LT, Guo P, Jiang HX, et al Proteomic analysis of Citrus sinensis roots and leaves in response to long-term magnesium-deficiency. BMC Genomics. 2015; 16: 253 10.1186/s12864-015-1462-z 25887480 PMC4383213

[48] Chen M, Li K, Li H, Song CP, Miao Y. The glutathione peroxidase gene family in gossypium hirsutum: genome-wide identification, classification, gene expression and functional analysis. Sci. Rep. 2017; 16(7): 44743. 10.1038/srep44743 PMC5353742 28300195

[49] Zheng JS, Yu CM, Chen P, Wang YZ, Tan LT, Chen JK, et al Characterization of a glutamine synthetase gene BnGS1-2 from ramie (*Boehmeria nivea* L. Gaud) and biochemical assays of BnGS1-2-over-expressing transgenic tobacco. Acta Physiol. Plant. 2015; 37: 1–10.

[50] Aguilar-Barragán A, Ochoa-Alejo N. Virus-induced silencing of MYB and WD40 transcription factor genes affects the accumulation of anthocyanins in chilli pepper fruit. Biol. Plant. 2014; 58: 567–574.

[51] Kaneko S, Kumazawa K, Masuda H, Henze A, Hofmann T. Molecular and sensory studies on the umami taste of Japanese green tea. J. Agric. Food Chem. 2006; 54: 2688–2694. 10.1021/jf0525232 16569062

[52] Deng WW, Ogita S, Ashihara H. Biosynthesis of theanine (γ-ethylamino-l-glutamic acid) in seedlings of Camellia sinensis. Phytochem. Lett. 2008; 1: 115–119.

[53] Kim JY, Kim WY, Kwak KJ, Oh SH, Han YS, Kang H. Glycine-rich RNA-binding proteins are functionally conserved in *Arabidopsis thaliana* and *Oryza sativa* during cold adaptation process. J. Exp. Bot. 2010; 61: 2317–2325. 10.1093/jxb/erq058 20231330 PMC2877889

[54] Kim JS, Jung HJ, Lee HJ, Kim K, Goh CH, Woo Y, et al Glycine-rich RNA-binding protein7 affects abiotic stress responses by regulating stomata opening and closing in Arabidopsis thaliana. Plant J. 2008; 55: 455–466. 10.1111/j.1365-313X.2008.03518.x 18410480

[55] Kim JY, Park SJ, Jang B, Jung CH, Ahn SJ, Goh CH, et al Functional characterization of a glycine-rich RNA-binding protein 2 in Arabidopsis thaliana under abiotic stress conditions. Plant J. 2007; 50: 439–451. 10.1111/j.1365-313X.2007.03057.x 17376161

[56] Cai G. Assembly and disassembly of plant microtubules: tubulin modifications and binding to MAPs. J. Exp. Bot. 2010; 61: 623–626. 10.1093/jxb/erp395 20080825

[57] Kopczak SD, Haas NA, Hussey PJ, Silflow CD, Snustad DP. The small genome of Arabidopsis contains at least six expressed alpha-tubulin genes. Plant Cell. 1992; 4: 539–547. 10.1105/tpc.4.5.539 1498608 PMC160151

