# Supplementary figures and images for: Proteomic analysis of tea plants (Camellia sinensis) with purple young shoots during leaf development
Source: PLoS One. 2017 May 16;12(5):e0177816. doi: 10.1371/journal.pone.0177816 (PMC5433784; doi:10.1371/journal.pone.0177816)

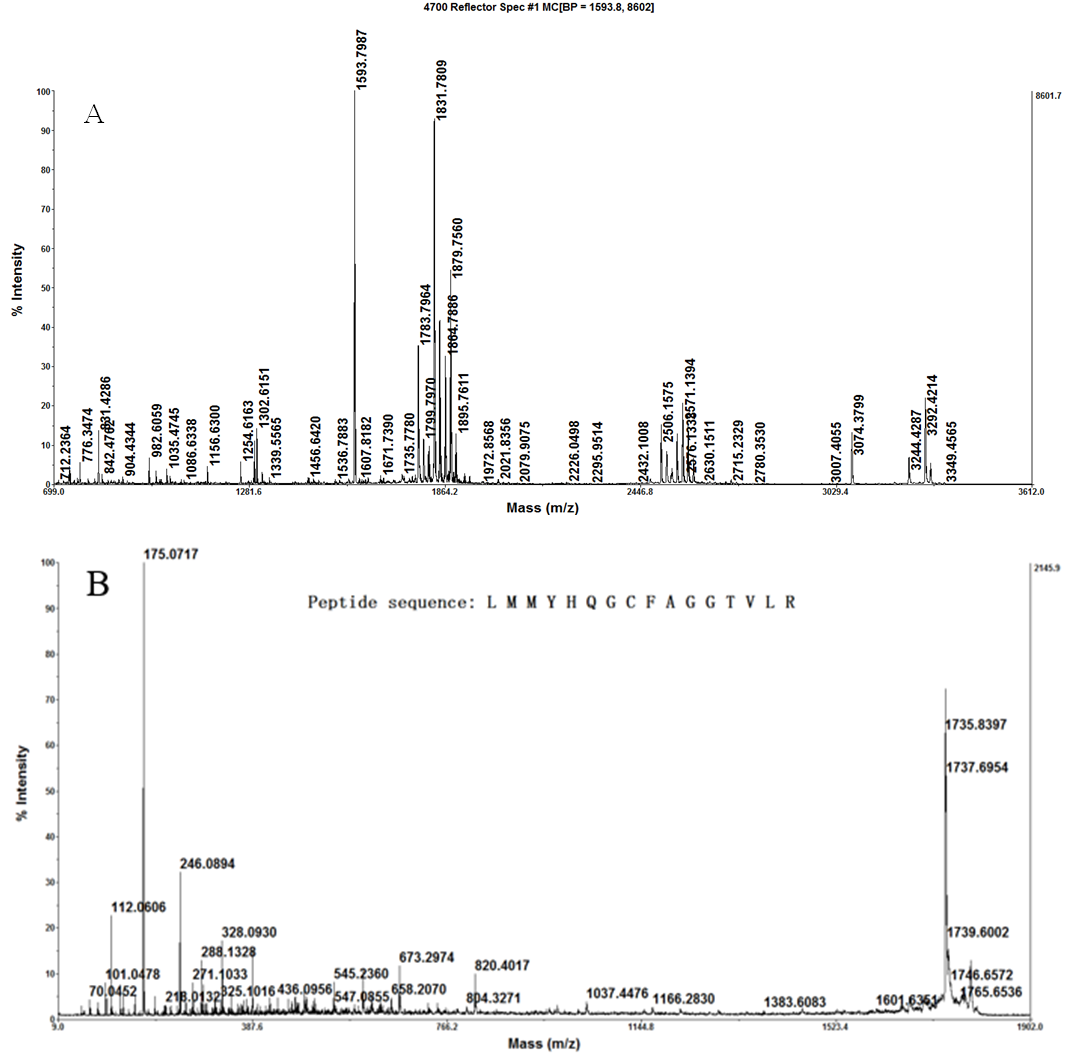

Supplement: S1 Fig — (A) Peptide mass fingerprinting of protein spot 8406; (B) MS/MS spectra of ion 1799.8, the corresponding peptide sequence are shown. (TIF) [file pone.0177816.s001.tif]

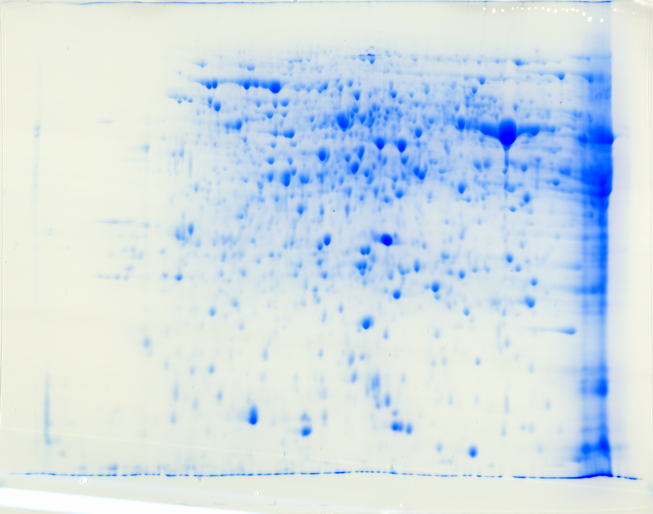
TPL1


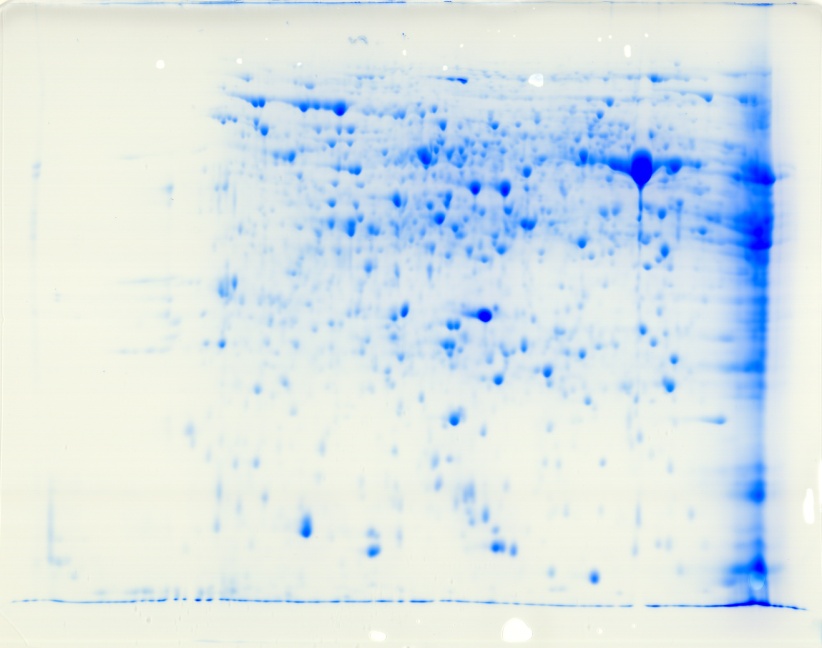
TPL2


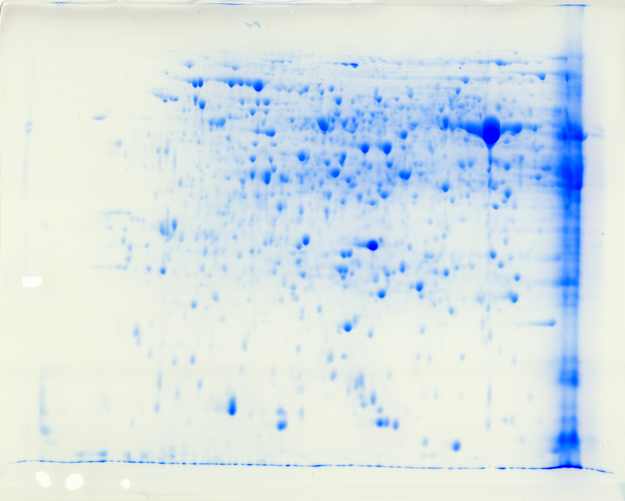
TPL3


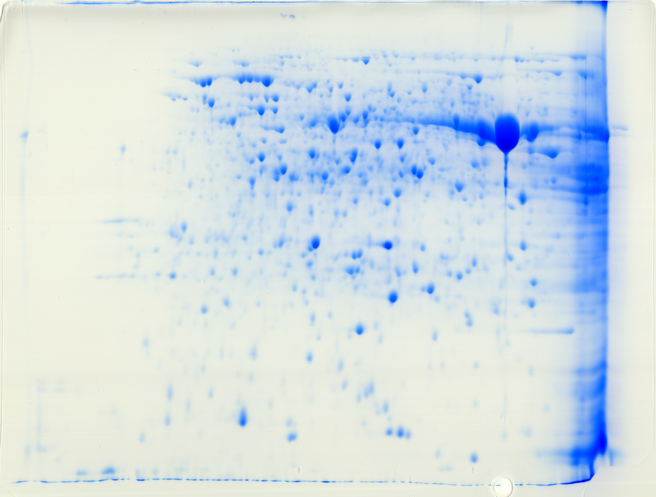
MGL1


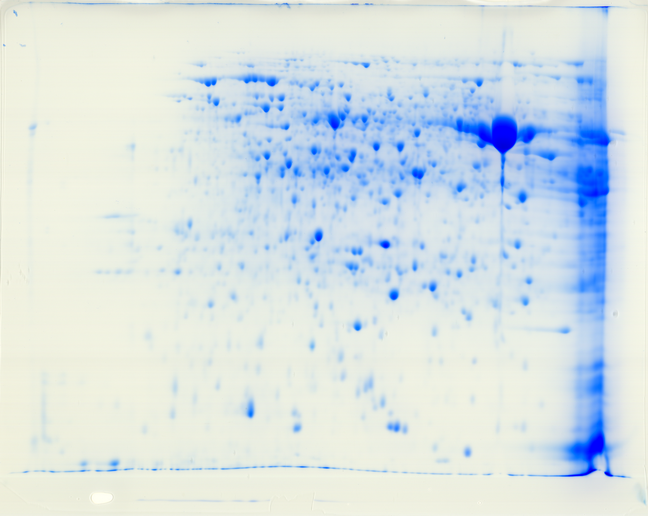
MGL2


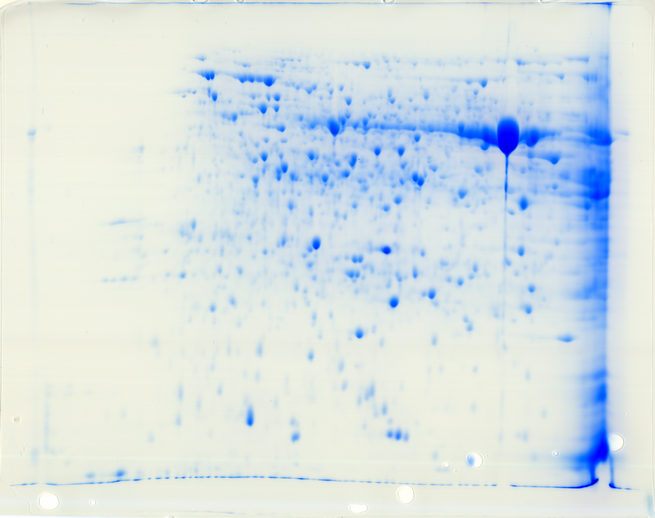
MGL3

Supplement: S2 Fig — (DOCX) [file pone.0177816.s002.docx]
