# Supplementary material for: Proteomic analysis of tea plants (Camellia sinensis) with purple young shoots during leaf development
Source: PLoS One. 2017 May 16;12(5):e0177816. doi: 10.1371/journal.pone.0177816 (PMC5433784; doi:10.1371/journal.pone.0177816)
Supplement: S3 Fig — (PDF) [file pone.0177816.s003.pdf]

Image Report For: color (Master)

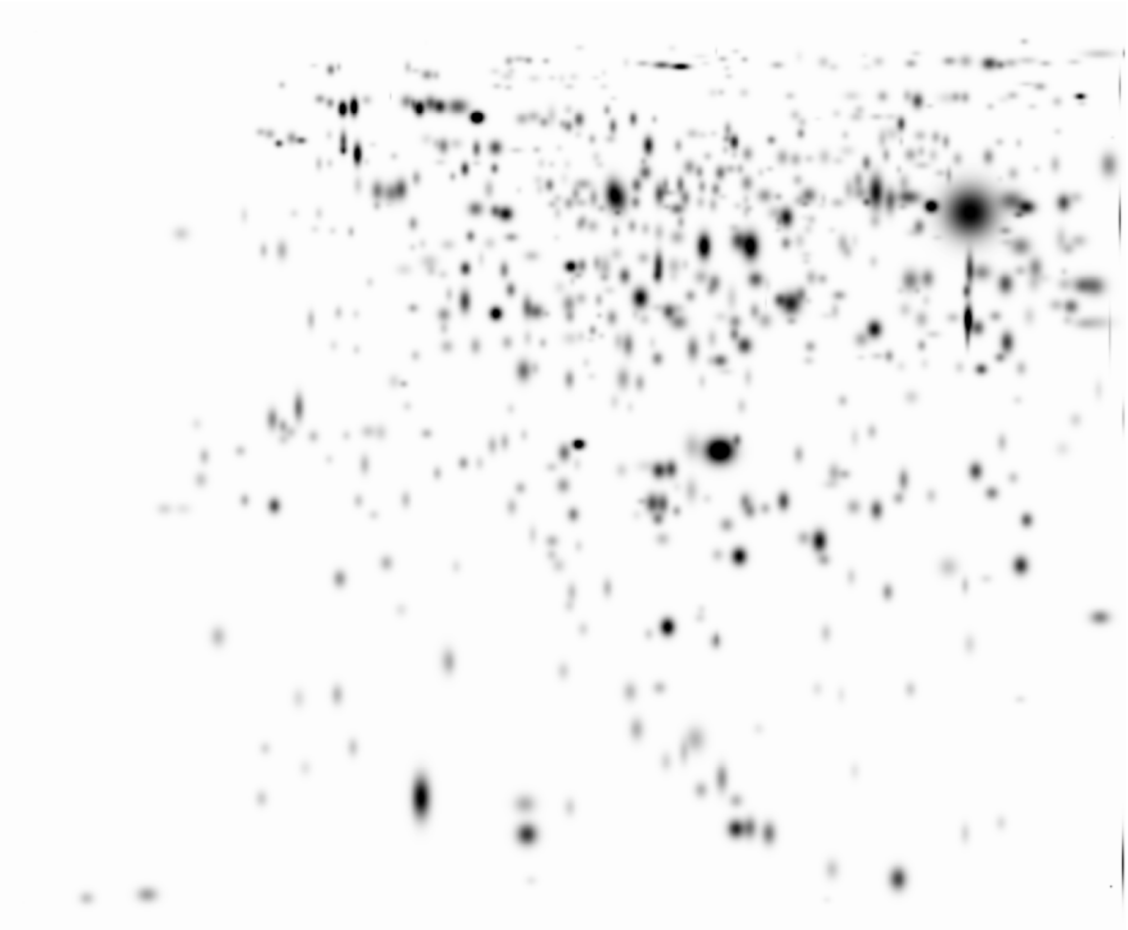

Description	N/A
Image Area(mm)	X: 235.7, Y: 194.5
Pixel size(um)	X: 105.8, Y: 105.8
Data Range	0.0 - 255.00 unknown
Total Spot Count	920
Valid Spot Count	665
Memory Size	79.63 Kb
Detect Log	<unknown>
Detect parameter set	<unknown>
