# Supplementary material for: Proteomic analysis of tea plants (Camellia sinensis) with purple young shoots during leaf development
Source: PLoS One. 2017 May 16;12(5):e0177816. doi: 10.1371/journal.pone.0177816 (PMC5433784; doi:10.1371/journal.pone.0177816)
Supplement: S1 Table — (DOCX) [file pone.0177816.s004.docx]

**S1 Table. Primer sequences used for quantitative real-time PCR**

| Protein Spot No. | Forward primers (5'-3') | Reverse primers (5'-3') |
| --- | --- | --- |
| 412 | ATCTCCCTCTCTCAACCACACG | CTCCTCCCCAACCTCTTTTAGC |
| 2405 | CTCCAGAGATTTCCACCTTC | ACCAATAACCTCAGACGACA |
| 2603 | CAAAAAGCATCTTCTGTGGC | TTTCCAAAAGGCGTGAGTG |
| 4106 | TCTCTCTCCTAAACTCTCATCG | AACTGCTTCCCATCATCCAACT |
| 8306 | GCTACCACTGATGTTGTTGAGG | GCAGCAGCATAACTCTCCAG |
| 8406 | CCCAAACCATTCTCCCCGATAG | CAACACGCACGCACTTGACATA |
| 9502 | AGCAGTTTATGAATGTCTCCGC | GCCCTTTGATTTCACCTGTTTC |
| 4304 | GTCGTCGTTCGTCGGAAAACT | TCATCGGGAGTCAAGAGCATG |
| 2206 | TGAAAGGAACTGGGACTGCTA | CCTCAATCTCATCAAGGGTGTA |
| 8304 | GTGGATGCTGCGTTCCCTCTT | GCATTGGTGTTGGCAGGGTTA |
| 6410 | ACAATAATTGGAGGCGGTGAC | CAGCAAAAGAAACTGAAGCAC |
| 4407 | ACCTCAGAAGCAAAGCAAGGA | TCACCAGCAGGAGTGTAAGCA |
| 2504 | AAGAGGATGCCGCTAATAACT | AAGGATAGATTGTGAAGCCAAG |
| 5116 | ATGGTCCGACTACTGTGAATGT | ATCCACTATTGTTGCCTCTGC |
| 5106 | AAAAGGTGTATGTTATGGGGAGG | AGACTGAAGTTGCCAAGGGTC |
| 18S rRNA | CCTGAGAAACGGCTACCACA | CACCAGACTTGCCCTCCA |
